# Dataset of genotoxic and cytotoxic effects on the pygmy mussel, *Xenostrobus securis*, from the highly urbanised Sydney Estuary, Australia: Relationships with metal bioaccumulation

**DOI:** 10.1016/j.dib.2020.105460

**Published:** 2020-03-20

**Authors:** Scott J. Markich

**Affiliations:** a Aquatic Solutions International, “Point Break”, North Narrabeen Beach, NSW 2101, Australia; b Department of Earth and Environmental Sciences, Macquarie University, North Ryde, NSW 2109, Australia

**Keywords:** Micronuclei, Lysosomal membrane stability, Haemocyte, DNA damage, Bioaccumulation, Estuarine, *Xenostrobus*, Mussel

## Abstract

This article contains a dataset of the genotoxic (DNA damage, via the micronucleus frequency test) and cytotoxic (lysosomal membrane stability (cellular integrity), via the neutral red retention test) effects on the pygmy mussel, *Xenostrobus securis* (Bivalvia: Mytilidae) from variably contaminated sites (primarily from cadmium (Cd), chromium (Cr), copper (Cu), lead (Pb) and zinc (Zn)) in the highly urbanized Sydney Estuary, south-eastern Australia. Data were collected 15 years apart (June 2004 and June 2019) to assess any change in (i) the “health” of mussels (based on the above two toxicity endpoints) and (ii) their metal contaminant status (measured as whole soft tissue concentrations of Cd, Cr, Cu, Pb and Zn). Linear relationships between both toxicity endpoints and metal concentrations in the whole soft tissue were also investigated. Multivariate statistical techniques, including principal components analysis, multidimensional scaling and cluster analysis, were also explored to reduce dimensional data, investigate patterns and assess similarities among study sites with respect to tissue metal concentrations and toxicity effects in *X. securis*. Enrichment factors were calculated by dividing the mean whole soft tissue metal concentration at each site in the Sydney Estuary, by its mean baseline metal concentration from near-pristine (reference) sites in the adjacent Hawkesbury Estuary. Salinity, pH, temperature, turbidity, dissolved oxygen and chlorophyll *a* were measured in the surface waters at each site

Specifications table This table needs re-formatting on the PROOF version

**Table:** 

Subject	Environmental Science
Specific subject area	Metal toxicity and accumulation in estuarine mussels
Type of data	Table Graph Figure
How data were acquired	Light/fluorescence microscopy (Olympus BX50), Microwave digestion (Milestone ETHOS 1), Inductively coupled plasma mass spectrometry (Agilent 4500 or 7900), Inductively coupled plasma atomic emission spectrometry (Varian Vista AX or Agilent 700), Water quality sonde (Yellow Springs Instruments 6000UPG or Horiba U52)
Data format	Raw Analyzed
Parameters for data collection	General physico-chemical (salinity, pH, temperature, turbidity, dissolved oxygen and chlorophyll a concentration) analyses of surface waters in the Sydney (highly urbanized) and Hawkesbury (near-pristine) estuaries in south-eastern Australia in June 2004 and June 2019. Measurement of the genotoxic (DNA damage) and cytotoxic (lysosomal membrane stability) effects on the intertidal estuarine mussel, *Xenostrobus securis*, as well as their whole soft tissue concentrations of key metals (cadmium, chromium, copper, lead and zinc).
Description of data collection	Haemolymph was extracted from mussels with a hypodermic syringe, mixed with buffered saline, placed on glass slides and air-dried in a dark humid chamber. To determine micronuclei frequency, haemocyte cells were fixed with 100% methanol, stained with 5% Giesma solution and mounted with Eukitt. Agranular cells were scored blind via light microscopy. To determine lysosomal membrane stability, haemocyte cells were stained with neutral red solution and incubated for 15, 30, 60, 90 and 120 min. Granular cells were scored blind from digitized images acquired via light microscopy. Haemocyte cell viability was assessed via fluorescence microscopy using differential acridine orange/ethidium bromide staining. Whole soft mussel tissue was dissected from the shell, oven-dried, homogenized with a mortar and pestle and microwave digested with a mixture of nitric acid and hydrogen peroxide.
Data source location	Region: Greater metropolitan Sydney Country: Australia GPS coordinates for collected data: 33°30–53′ S to 151°01–18′ E
Data accessibility	With the article (including supplementary material)

## Value of the Data

• Data provide a quality-assured temporal assessment (2004 versus 2019) of the bioavailability and toxicity of key metal contaminants in the Sydney Estuary, whereby changes in metal contaminant status can be detected to better manage and protect marine ecosystems.
• Data will be of benefit to coastal pollution researchers, managers, planners and policy makers, and ultimately, the public through greater recreational enjoyment of an iconic natural resource (Sydney Harbor waterways).
• First genotoxic (DNA damage, using micronuclei frequency) and cytotoxic (cellular integrity, using lysosomal membrane stability) field data worldwide for the estuarine (mytilid) mussel, *Xenostrobus securis* – serving as a benchmark for further investigation.
• One of only few studies worldwide to report quantitative (rather than traditional semi-quantitative) field data for lysosomal membrane stability in mytilids.
• Data permit univariate and multivariate relationships to be determined among whole soft tissue concentrations of cadmium, chromium, copper, lead and zinc and toxicity effects (micronuclei frequency or lysosomal membrane stability) in *X. securis*.

## 1 Data description

**Fig. 1:**
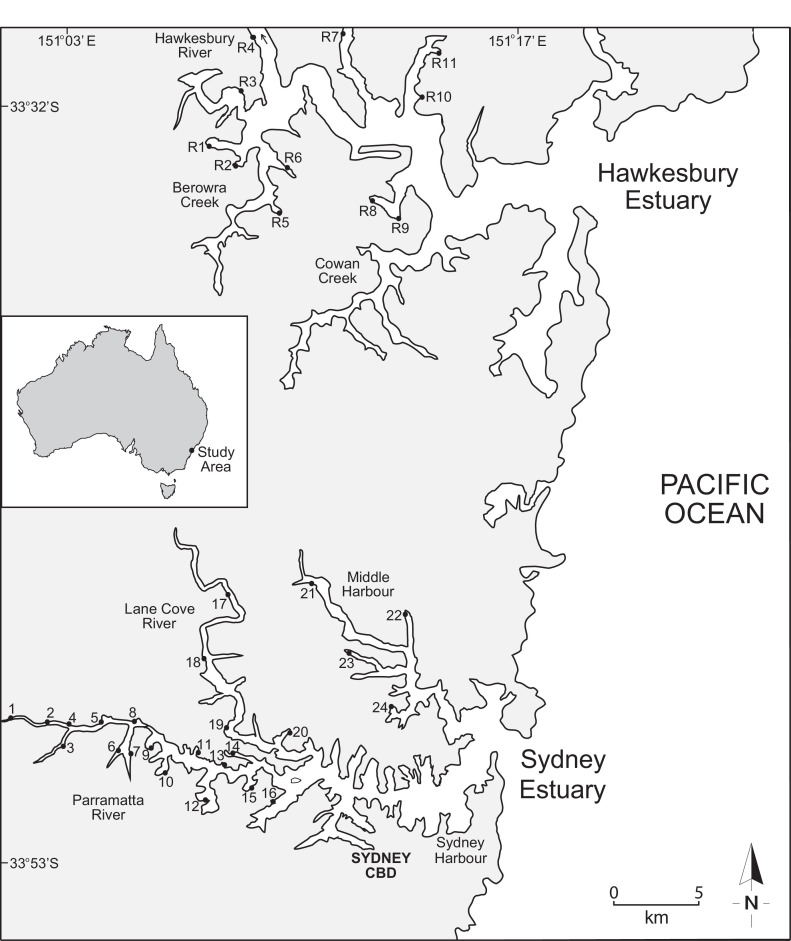
Location map showing the study sites in the Hawkesbury and Sydney estuaries, south-eastern Australia (inset). Global positioning system (GPS) coordinates for each site are provided in Markich and Jeffree [2].

**Fig. 2:**
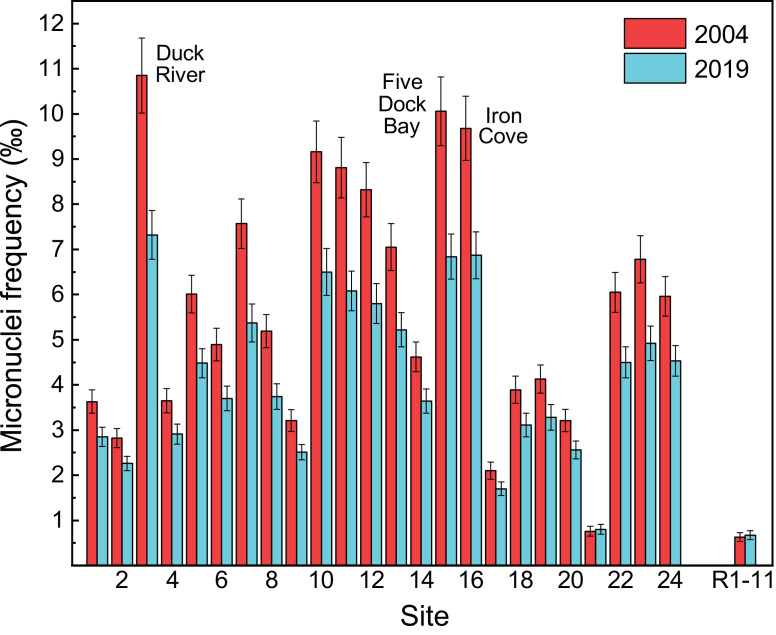
Mean micronuclei frequency (‰MF) (and 84% confidence interval) for *Xenostrobus securis* from variably contaminated sites (1–24) in the Sydney Estuary, relative to near-pristine (reference) sites (R1−11) in the Hawkesbury Estuary, for two sampling events (2004 and 2019). Non-overlapping confidence intervals denote significant differences (*p* ≤ 0.05) among sites or between sampling times. There was a 30% decrease (improvement) in ‰MF between 2004 and 2019 for the six most contaminated sites (see Fig. 5 and Table S6). See Fig. 1 for site locations and Table S2 for raw data.

**Fig. 3:**
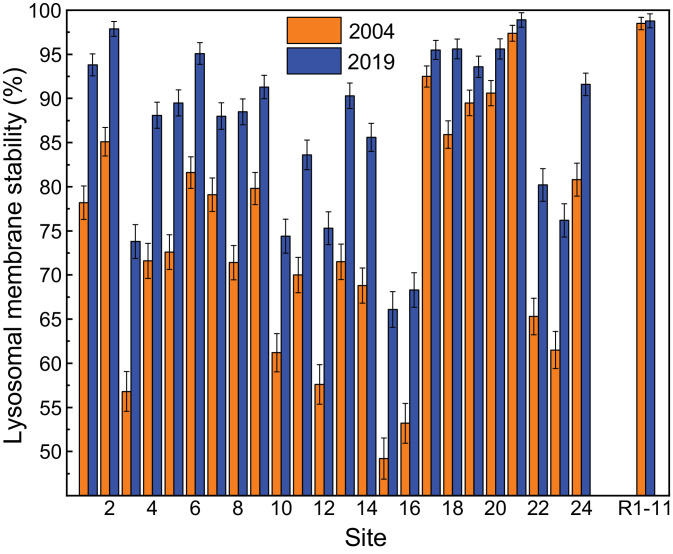
Mean percentage lysosomal membrane stability (%LMS) (and 84% confidence interval) for *Xenostrobus securis* from variably contaminated sites (1–24) in the Sydney Estuary, relative to near-pristine (reference) sites (R1−11) in the Hawkesbury Estuary, for two sampling events (2004 and 2019). Non-overlapping confidence intervals denote significant differences (*p* ≤ 0.05) among sites or between sampling times. There was a 16% increase (improvement) in %LMS between 2004 and 2019 for the six most contaminated sites (see Fig. 5 and Table S6). See Fig. 1 for site locations and Table S2 for raw data.

Variably contaminated sites (1‒24) in the highly urbanised Sydney Estuary, and near-pristine (reference) sites (R1‒11) in the adjacent Hawkesbury Estuary, in southern-eastern Australia, are presented in Fig. 1. The general physico-chemistry (salinity, pH, temperature, turbidity, dissolved oxygen and chlorophyll *a* concentration) of surface waters in the Hawkesbury and Sydney estuaries, for both the 2004 and 2019 sampling events, is provided in Tables 1 and 2, respectively. The mean micronuclei frequency (‰MF) and percentage lysosomal membrane stability (%LMS) in haemocytes of wild *Xenostrobus securis* (pygmy mussel) from variably contaminated sites (1–24) in the Sydney Estuary, relative to near-pristine (reference) sites (R1−11) in the Hawkesbury Estuary, for the two sampling events (2004 and 2019), is presented in Figs. 2 and 3, respectively (raw data are provided in Appendix A; Table S1 for the Hawkesbury Estuary and Table S2 for the Sydney Estuary). The ‰MF in haemocytes of wild mussels (Mytilidae) from minimally-contaminated (reference) sites worldwide (salinity >25‰), is given in Appendix A (Table S3).

**Table 1:** Physico-chemistry of surface water for near-pristine (reference) sites in the Hawkesbury Estuary, for two sampling events (2004 and 2019).

Site^a^	Salinity (‰)		pH		Temperature (°C)		Turbidity (NTU)		Dissolved oxygen (% saturation)		Chlorophyll *a* (µg/L)	
	2004	2019	2004	2019	2004	2019	2004	2019	2004	2019	2004	2019
R1	27.4 ± 0.2^b^	27.6 ± 0.2	7.62 ± 0.03	7.64 ± 0.03	16.0 ± 0.2	16.1 ± 0.2	8.2 ± 0.4	8.0 ± 0.4	80.1 ± 3.6	77.5 ± 3.4	4.1 ± 0.4	4.3 ± 0.3
R2	27.5 ± 0.2	27.4 ± 0.2	7.64 ± 0.03	7.62 ± 0.03	16.1 ± 0.2	16.2 ± 0.2	8.1 ± 0.4	7.9 ± 0.4	79.4 ± 3.6	80.2 ± 3.2	4.3 ± 0.4	4.4 ± 0.4
R3	27.6 ± 0.2	27.4 ± 0.2	7.63 ± 0.03	7.63 ± 0.03	15.9 ± 0.2	16.0 ± 0.2	8.5 ± 0.5	8.2 ± 0.5	76.4 ± 3.2	79.4 ± 3.2	4.7 ± 0.3	4.8 ± 0.4
R4	27.5 ± 0.2	27.4 ± 0.2	7.64 ± 0.03	7.65 ± 0.03	15.8 ± 0.3	15.9 ± 0.3	8.1 ± 0.4	7.8 ± 0.4	81.3 ± 3.6	78.4 ± 3.2	4.2 ± 0.3	4.3 ± 0.3
R5	25.8 ± 0.2	25.9 ± 0.2	7.58 ± 0.04	7.60 ± 0.04	15.7 ± 0.3	15.8 ± 0.3	8.8 ± 0.6	8.5 ± 0.5	74.7 ± 3.6	77.2 ± 3.6	5.4 ± 0.3	5.2 ± 0.3
R6	26.8 ± 0.1	27.0 ± 0.2	7.61 ± 0.04	7.63 ± 0.04	16.1 ± 0.2	16.2 ± 0.2	8.6 ± 0.5	8.3 ± 0.6	80.8 ± 3.2	82.6 ± 3.2	4.4 ± 0.4	4.6 ± 0.3
R7	28.9 ± 0.2	29.0 ± 0.1	7.78 ± 0.03	7.79 ± 0.03	16.1 ± 0.2	16.2 ± 0.2	7.8 ± 0.5	7.5 ± 0.4	76.4 ± 3.6	78.6 ± 3.2	4.1 ± 0.3	4.2 ± 0.3
R8	33.0 ± 0.1	33.0 ± 0.1	7.95 ± 0.03	7.96 ± 0.03	16.3 ± 0.2	16.4 ± 0.2	5.7 ± 0.4	5.3 ± 0.4	83.6 ± 3.2	81.9 ± 3.6	3.4 ± 0.3	3.5 ± 0.3
R9	33.4 ± 0.2	33.3 ± 0.1	7.99 ± 0.03	7.98 ± 0.03	16.3 ± 0.2	16.4 ± 0.2	5.1 ± 0.4	4.8 ± 0.4	85.2 ± 3.2	82.8 ± 3.6	3.2 ± 0.3	3.4 ± 0.3
R10	32.5 ± 0.1	32.7 ± 0.2	7.90 ± 0.03	7.92 ± 0.03	16.3 ± 0.2	16.4 ± 0.2	5.9 ± 0.4	5.6 ± 0.4	83.7 ± 3.6	84.9 ± 3.2	3.4 ± 0.2	3.3 ± 0.3
R11	30.4 ± 0.2	30.6 ± 0.2	7.83 ± 0.03	7.86 ± 0.03	16.2 ± 0.2	16.3 ± 0.2	8.3 ± 0.5	8.0 ± 0.5	77.4 ± 3.6	79.1 ± 3.2	4.0 ± 0.3	4.3 ± 0.3
**Mean**	**29.1** **±** **0.2**	**29.2** **±** **0.2**	**7.74** **±** **0.04**	**7.75** **±** **0.04**	**16.1** **±** **0.3**	**16.2** **±** **0.3**	**7.6** **±** **0.6**	**7.3** **±** **0.6**	**79.9** **±** **3.6**	**80.2** **±** **3.6**	**4.1** **±** **0.4**	**4.2** **±** **0.4**

a See Fig. 1 for location map.

b Mean ± 84% confidence limit (*n* = 2). For each variable, there was no significant (*p* ≤ 0.05) difference between 2004 and 2019.

**Table 2:** Physico-chemistry of surface water for variably contaminated sites in the Sydney Estuary, for two sampling events (2004 and 2019).

Site^a^	Salinity (‰)		pH		Temperature ( °C)		Turbidity (NTU)		Dissolved oxygen (% saturation)		Chlorophyll *a* (µg/L)	
	2004	2019	2004	2019	2004	2019	2004	2019	2004	2019	2004	2019
1	26.0 ± 0.3^b^	26.3 ± 0.3	7.55 ± 0.04	7.58 ± 0.04	15.5 ± 0.3	15.7 ± 0.3	13.1 ± 0.8	12.7 ± 0.7	73.0 ± 3.6	75.1 ± 3.6	5.9 ± 0.5	6.2 ± 0.4
2	27.5 ± 0.3	27.6 ± 0.3	7.62 ± 0.04	7.64 ± 0.04	15.6 ± 0.3	15.8 ± 0.3	12.6 ± 0.8	12.1 ± 0.7	75.2 ± 3.6	77.3 ± 3.6	5.6 ± 0.5	5.4 ± 0.4
3	27.6 ± 0.2	27.4 ± 0.3	7.62 ± 0.04	7.65 ± 0.04	15.7 ± 0.2	15.8 ± 0.2	12.8 ± 0.8	12.0 ± 0.7	75.8 ± 3.6	77.9 ± 3.6	5.6 ± 0.5	5.7 ± 0.5
4	28.5 ± 0.2	28.3 ± 0.2	7.75 ± 0.03	7.73 ± 0.03	15.8 ± 0.3	15.9 ± 0.3	11.6 ± 0.8	11.2 ± 0.7	77.1 ± 3.2	79.5 ± 3.2	4.7 ± 0.4	5.0 ± 0.4
5	29.3 ± 0.2	29.2 ± 0.2	7.80 ± 0.03	7.78 ± 0.03	15.9 ± 0.3	16.0 ± 0.3	10.6 ± 0.8	10.0 ± 0.8	76.3 ± 3.6	77.9 ± 3.2	4.2 ± 0.5	4.4 ± 0.4
6	30.6 ± 0.2	30.3 ± 0.2	7.83 ± 0.03	7.81 ± 0.03	16.0 ± 0.2	16.2 ± 0.2	7.5 ± 0.7	7.7 ± 0.8	77.9 ± 3.2	78.8 ± 3.2	3.7 ± 0.4	3.9 ± 0.5
7	30.5 ± 0.2	30.2 ± 0.2	7.84 ± 0.03	7.82 ± 0.03	16.1 ± 0.2	16.2 ± 0.2	8.4 ± 0.7	8.0 ± 0.7	78.2 ± 3.2	78.9 ± 3.2	3.8 ± 0.4	4.1 ± 0.4
8	30.8 ± 0.2	30.6 ± 0.2	7.86 ± 0.03	7.83 ± 0.03	16.1 ± 0.2	16.3 ± 0.2	9.6 ± 0.8	9.1 ± 0.8	80.0 ± 3.2	81.9 ± 3.6	3.9 ± 0.4	4.3 ± 0.4
9	31.6 ± 0.2	31.5 ± 0.2	7.89 ± 0.03	7.87 ± 0.03	16.1 ± 0.2	16.3 ± 0.2	7.4 ± 0.7	6.9 ± 0.6	79.2 ± 3.2	80.8 ± 3.2	3.5 ± 0.4	3.7 ± 0.4
10	31.9 ± 0.2	31.6 ± 0.2	7.88 ± 0.03	7.90 ± 0.03	16.2 ± 0.2	16.4 ± 0.2	7.2 ± 0.6	6.8 ± 0.6	78.8 ± 3.6	80.2 ± 3.2	3.3 ± 0.4	3.6 ± 0.4
11	32.4 ± 0.2	32.3 ± 0.2	7.89 ± 0.03	7.88 ± 0.03	16.2 ± 0.2	16.4 ± 0.2	6.9 ± 0.6	6.4 ± 0.7	80.2 ± 3.2	81.8 ± 3.2	3.0 ± 0.5	3.3 ± 0.4
12	32.8 ± 0.2	32.7 ± 0.2	7.95 ± 0.03	7.92 ± 0.03	16.2 ± 0.2	16.3 ± 0.2	6.6 ± 0.6	6.0 ± 0.6	81.4 ± 3.2	82.8 ± 3.2	2.8 ± 0.4	3.0 ± 0.5
13	33.3 ± 0.2	33.2 ± 0.1	7.97 ± 0.03	7.95 ± 0.03	16.3 ± 0.2	16.4 ± 0.2	6.6 ± 0.5	6.2 ± 0.5	81.9 ± 3.2	83.3 ± 3.2	2.8 ± 0.5	3.1 ± 0.4
14	32.7 ± 0.2	32.6 ± 0.2	7.93 ± 0.03	7.93 ± 0.03	16.2 ± 0.2	16.3 ± 0.2	7.7 ± 0.6	8.2 ± 0.6	77.0 ± 3.6	79.1 ± 3.2	3.4 ± 0.5	3.6 ± 0.5
15	33.4 ± 0.1	33.2 ± 0.2	7.96 ± 0.03	7.94 ± 0.03	16.3 ± 0.2	16.4 ± 0.2	6.6 ± 0.5	6.3 ± 0.5	81.8 ± 3.2	82.8 ± 3.2	2.7 ± 0.5	2.9 ± 0.4
16	33.6 ± 0.2	33.5 ± 0.1	7.99 ± 0.03	7.97 ± 0.03	16.3 ± 0.2	16.4 ± 0.2	6.5 ± 0.4	6.1 ± 0.5	83.1 ± 3.6	84.6 ± 3.2	2.5 ± 0.5	2.7 ± 0.5
17	26.5 ± 0.2	26.6 ± 0.3	7.58 ± 0.04	7.60 ± 0.04	15.7 ± 0.3	15.7 ± 0.3	12.9 ± 0.7	12.0 ± 0.6	74.1 ± 3.6	76.5 ± 3.6	5.8 ± 0.5	6.1 ± 0.4
18	28.7 ± 0.2	28.8 ± 0.2	7.76 ± 0.03	7.77 ± 0.03	15.8 ± 0.2	15.9 ± 0.2	10.8 ± 0.7	10.3 ± 0.7	75.4 ± 3.6	78.1 ± 3.6	4.9 ± 0.5	5.0 ± 0.4
19	31.9 ± 0.2	32.0 ± 0.2	7.89 ± 0.03	7.90 ± 0.03	16.1 ± 0.2	16.2 ± 0.2	9.6 ± 0.6	9.1 ± 0.5	78.9 ± 3.6	79.9 ± 3.2	3.9 ± 0.5	4.1 ± 0.5
20	32.7 ± 0.2	32.8 ± 0.2	7.94 ± 0.03	7.94 ± 0.03	16.2 ± 0.2	16.3 ± 0.2	7.6 ± 0.6	7.4 ± 0.5	79.5 ± 3.2	82.1 ± 3.2	3.1 ± 0.5	3.4 ± 0.5
21	33.1 ± 0.1	33.2 ± 0.2	7.96 ± 0.03	7.97 ± 0.03	16.2 ± 0.2	16.3 ± 0.2	7.1 ± 0.6	6.6 ± 0.5	82.8 ± 3.2	84.7 ± 3.2	3.0 ± 0.4	3.2 ± 0.5
22	33.4 ± 0.2	33.5 ± 0.1	7.98 ± 0.03	7.99 ± 0.03	16.3 ± 0.2	16.4 ± 0.2	6.6 ± 0.5	6.2 ± 0.5	83.8 ± 3.2	85.3 ± 3.2	2.8 ± 0.5	2.9 ± 0.4
23	33.6 ± 0.2	33.7 ± 0.1	8.00 ± 0.03	7.98 ± 0.03	16.3 ± 0.2	16.4 ± 0.2	7.6 ± 0.6	7.3 ± 0.5	84.5 ± 3.2	87.1 ± 3.2	2.6 ± 0.5	2.8 ± 0.4
24	33.8 ± 0.1	33.9 ± 0.1	8.02 ± 0.03	8.00 ± 0.03	16.3 ± 0.2	16.4 ± 0.2	7.4 ± 0.4	7.0 ± 0.5	85.3 ± 3.6	86.9 ± 3.2	2.5 ± 0.5	2.7 ± 0.4
**Mean**	**31.1** **±** **0.3**	**31.0** **±** **0.3**	**7.85** **±** **0.04**	**7.85** **±** **0.04**	**16.0** **±** **0.3**	**16.2** **±** **0.3**	**8.5** **±** **0.8**	**8.1** **±** **0.8**	**79.1** **±** **3.6**	**80.9** **±** **3.6**	**3.7** **±** **0.5**	**3.9** **±** **0.5**

a See Fig. 1 for location map.

b Mean ± 84% confidence limit (*n* = 2). For each variable, there was no significant (*p* ≤ 0.05) difference between 2004 and 2019.

**Fig. 4:**
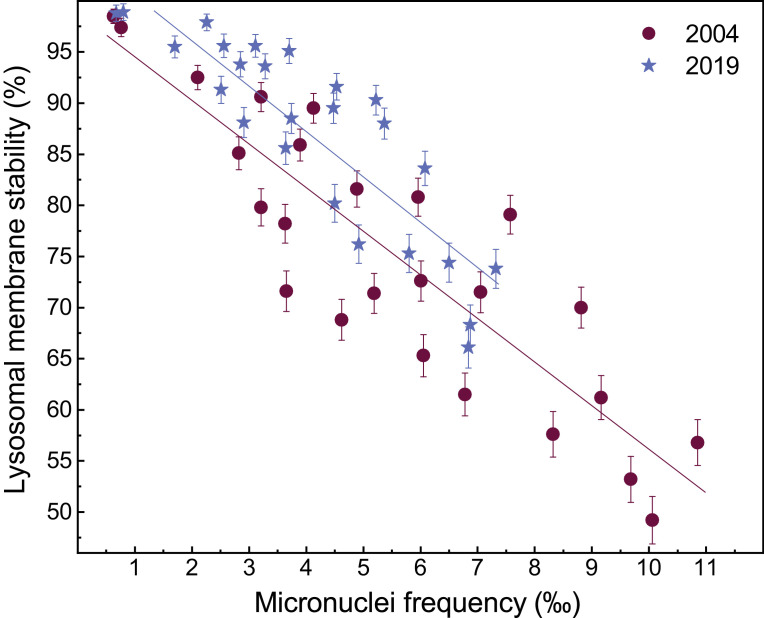
Inverse linear relationships between percentage lysosomal membrane stability (%LMS) and micronuclei frequency (‰MF) in haemocytes of *Xenostrobus securis* from the Sydney/Hawkesbury estuaries, for two sampling events (2004 and 2019). Data represent mean values and their 84% confidence interval. Linear regression equations for 2004 and 2019 are %LMS = −4.22(‰MF) + 98.3 (*r*^2^ = 0.76, *p* <0.01) and %LMS = −4.54(‰MF) + 105 (*r*^2^ = 0.74, *p* <0.01), respectively. See Table S2 for raw data.

The results of factorial (two-way) analysis of variance, with site (1−24 and R1−11) and sampling time (2004 or 2019) as independent variables and (i) ‰MF and (ii) %LMS in the haemocytes of *X. securis* as the dependent variable, are provided in Appendix A (Table S4 for ‰MF and Table S5 for %LMS). Inverse linear relationships between %LMS and ‰MF in the haemocytes of *X. securis* from the Sydney and Hawkesbury estuaries, for two sampling events (2004 and 2019), are presented in Fig. 4 (raw data are given in Appendix A; Tables S1 and S2).

**Fig. 5:**
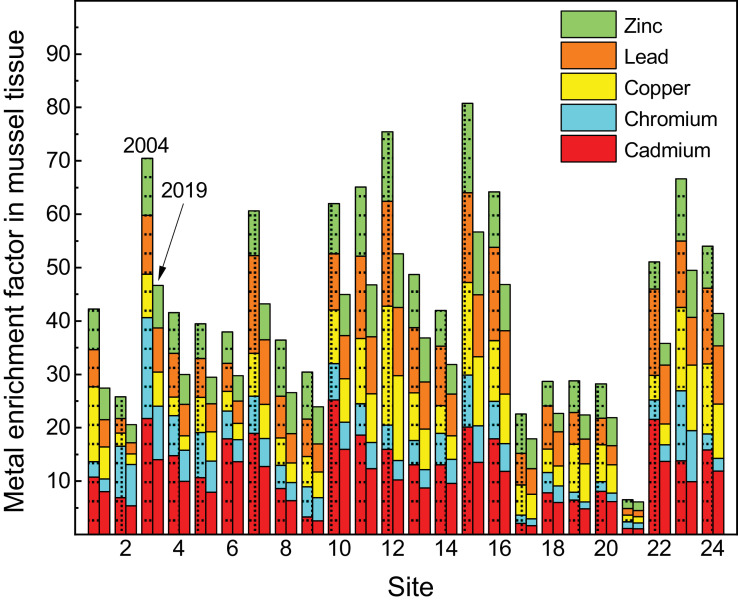
Mean enrichment factors of cadmium, chromium, copper, lead and zinc in the whole soft tissue of *Xenostrobus securis* from variably contaminated sites (1–24) in the Sydney Estuary, for two sampling events (2004 and 2019). See Fig. 1 for site locations and Table S6 for raw data.

The whole soft tissue concentrations (µg/dry weight) of cadmium (Cd), chromium (Cr), copper (Cu), lead (Pb) and zinc (Zn), the five key contaminants in the Sydney Estuary [1], in *X. securis*, from variably contaminated sites (1 − 24) in the Sydney Estuary, relative to reference sites (R1−11) in the Hawkesbury Estuary, for the 2004 and 2019 sampling events, are presented in Table 3. The mean enrichment factors (see Section 2.7 for definition) of Cd, Cr, Cu. Pb and Zn in the whole soft tissue of *X. securis* from variably contaminated sites in the Sydney Estuary, are presented in Fig. 5 (raw data are given in Appendix A; Table S6). Linear regression equations and coefficients of determination (*r*^2^) for Cd, Cr, Cu, Pb and Zn concentrations in the whole soft tissue of *X. securis* from the Sydney and Hawkesbury estuaries, as a function of ‰MF or %LMS, for two sampling events (2004 and 2019), are provided in Table 4. Positive linear relationships were previously established between Cd, Cr, Cu, Pb and Zn concentrations in the whole soft tissues of *X. securis* and those in (i) filtered (0.2 µm) surface water, (ii) suspended sediments and (iii) surface sediment [1].

**Table 3:** Cadmium, chromium, copper, lead and zinc concentrations in the whole soft tissue of *Xenostrobus securis* (µg/g dry weight) from variably contaminated sites (1−24) in the Sydney Estuary, relative to near-pristine (reference) sites (R1−11) in the Hawkesbury Estuary, for two sampling events (2004 and 2019).

Site^a^	Cadmium		Chromium		Copper		Lead		Zinc	
	2004	2019	2004	2019	2004	2019	2004	2019	2004	2019
1	7.42 ± 0.74^b^	5.79 ± 0.53	3.32 ± 0.31	2.60 ± 0.27	55.7 ± 5.51	39.1 ± 3.85	10.6 ± 1.01	7.81 ± 0.79	369 ± 38.2	277 ± 27.2
2	4.75 ± 0.46	3.86 ± 0.40	11.1 ± 1.08	8.45 ± 0.86	16.9 ± 1.63	13.1 ± 1.29	4.12 ± 0.43	3.21 ± 0.33	195 ± 19.1	156 ± 15.2
3^c^	15.0 ± 1.42	10.1 ± 1.03	15.7 ± 1.49	11.0 ± 1.02	55.8 ± 5.41	41.6 ± 4.36	16.7 ± 1.62	12.7 ± 1.21	517 ± 52.9	372 ± 36.5
4	10.2 ± 0.95	7.18 ± 0.70	8.62 ± 0.88	6.31 ± 0.62	23.7 ± 2.32	17.8 ± 1.86	12.4 ± 1.25	9.04 ± 0.90	369 ± 38.5	262 ± 27.2
5	7.32 ± 0.71	5.71 ± 0.56	9.76 ± 0.95	6.35 ± 0.60	44.9 ± 4.41	35.9 ± 3.43	11.0 ± 1.05	8.04 ± 0.78	317 ± 30.2	234 ± 24.3
6	12.4 ± 1.18	9.80 ± 0.96	6.00 ± 0.59	4.51 ± 0.44	25.2 ± 2.63	20.1 ± 1.94	7.98 ± 0.82	6.38 ± 0.65	285 ± 27.3	222 ± 21.5
7	13.1 ± 1.26	9.16 ± 0.90	7.98 ± 0.83	5.75 ± 0.58	54.7 ± 4.51	41.7 ± 4.03	27.7 ± 2.68	18.6 ± 1.74	406 ± 41.3	315 ± 30.1
8	5.95 ± 0.57	4.58 ± 0.46	5.01 ± 0.49	3.65 ± 0.37	35.0 ± 3.53	24.2 ± 2.39	11.9 ± 1.13	8.42 ± 0.82	507 ± 48.5	359 ± 33.5
9	2.28 ± 0.22	1.85 ± 0.19	6.46 ± 0.62	4.72 ± 0.44	38.7 ± 3.81	31.4 ± 3.06	10.7 ± 1.08	8.09 ± 0.81	426 ± 43.4	324 ± 33.6
10	17.4 ± 1.68	11.5 ± 1.06	7.86 ± 0.82	5.58 ± 0.58	68.9 ± 6.97	53.0 ± 5.24	15.9 ± 1.63	12.4 ± 1.23	454 ± 43.6	360 ± 37.6
11^c^	12.9 ± 1.26	8.87 ± 0.89	6.75 ± 0.65	5.40 ± 0.55	83.5 ± 8.46	59.3 ± 5.86	23.4 ± 2.28	16.4 ± 1.55	625 ± 63.5	456 ± 44.3
12^c^	11.0 ± 1.01	7.38 ± 0.72	5.16 ± 0.48	3.92 ± 0.41	153 ± 15.1	104 ± 10.1	29.7 ± 2.79	19.6 ± 1.89	629 ± 61.1	472 ± 45.3
13	9.01 ± 0.90	6.29 ± 0.64	5.19 ± 0.55	3.74 ± 0.38	61.3 ± 6.32	49.7 ± 4.77	18.5 ± 1.81	13.5 ± 1.31	481 ± 47.3	385 ± 39.6
14	9.02 ± 0.88	6.86 ± 0.70	6.74 ± 0.64	4.99 ± 0.48	35.7 ± 3.42	28.5 ± 2.81	16.9 ± 1.72	12.0 ± 1.20	324 ± 33.5	259 ± 24.3
15^c^	13.9 ± 1.35	9.73 ± 1.02	11.2 ± 1.02	7.48 ± 0.71	119 ± 11.4	84.6 ± 8.26	25.4 ± 2.43	17.8 ± 1.70	810 ± 76.8	551 ± 52.6
16^c^	12.4 ± 1.25	8.52 ± 0.84	8.06 ± 0.82	5.64 ± 0.57	78.0 ± 7.89	60.8 ± 6.21	26.4 ± 2.61	18.2 ± 1.76	505 ± 50.3	406 ± 40.2
17	1.45 ± 0.16	1.19 ± 0.13	1.74 ± 0.18	1.43 ± 0.15	38.7 ± 3.56	29.4 ± 3.02	9.01 ± 0.90	7.39 ± 0.72	355 ± 34.3	262 ± 25.3
18	5.39 ± 0.53	4.32 ± 0.41	4.32 ± 0.44	3.41 ± 0.33	30.3 ± 2.95	24.3 ± 2.35	12.3 ± 1.16	9.81 ± 1.00	220 ± 22.6	161 ± 16.8
19	4.43 ± 0.43	3.45 ± 0.36	1.74 ± 0.19	1.46 ± 0.15	61.4 ± 5.89	46.6 ± 4.52	9.02 ± 0.92	7.03 ± 0.68	288 ± 29.2	213 ± 20.1
20	5.56 ± 0.54	4.42 ± 0.46	2.14 ± 0.22	1.72 ± 0.17	47.0 ± 4.69	34.8 ± 3.41	7.52 ± 0.76	5.48 ± 0.53	313 ± 30.2	247 ± 23.6
21	0.79 ± 0.086	0.76 ± 0.082	1.40 ± 0.15	1.16 ± 0.12	8.68 ± 0.92	7.81 ± 0.86	1.82 ± 0.19	1.75 ± 0.18	81.6 ± 8.53	76.6 ± 8.06
22	14.9 ± 1.47	9.85 ± 0.95	4.23 ± 0.43	3.38 ± 0.32	31.3 ± 3.07	25.7 ± 2.51	24.5 ± 2.31	16.9 ± 1.62	246 ± 24.3	189 ± 18.3
23^c^	9.51 ± 0.93	7.13 ± 0.68	15.1 ± 1.45	10.4 ± 0.99	107 ± 10.5	80.2 ± 7.89	18.8 ± 1.82	13.7 ± 1.35	565 ± 57.6	412 ± 40.3
24	10.9 ± 1.04	8.53 ± 0.87	3.42 ± 0.33	2.60 ± 0.26	90.0 ± 9.12	66.6 ± 6.53	21.4 ± 2.04	16.7 ± 1.62	382 ± 35.9	283 ± 27.6
R1−11	0.69 ± 0.076	0.72 ± 0.080	1.15 ± 0.12	1.09 ± 0.12	6.84 ± 0.76	6.54 ± 0.75	1.51 ± 0.16	1.53 ± 0.16	48.4 ± 5.16	46.8 ± 5.05

a See Fig. 1 for location map.

b Mean ± 84% confidence limit (*n* = 20).

c There was a significant (*p* ≤ 0.05) decrease in the mean tissue concentration of cadmium (35%), chromium (26%), copper (24%), lead (33%) and zinc (27%) between 2004 and 2019, for the six most contaminated sites (based on combined metal enrichment factors – see Fig. 5 and Table S6).

**Table 4:** Linear regression equations and coefficients of determination (*r*^2^) for cadmium, chromium, copper, lead and zinc concentrations in the whole soft tissue of *Xenostrobus securis* (µg/g dry weight) from the Sydney/Hawkesbury estuaries, as a function of micronuclei frequency (‰) or lysosomal membrane stability (%), for two sampling events (2004 and 2019)^a^.

Metal	Micronuclei frequency (‰)^b^				Lysosomal membrane stability (%)^c^			
	2004		2019		2004		2019	
	Equation	*r*^2^	Equation	*r*^2^	Equation	*r*^2^	Equation	*r*^2^
Cadmium (Cd)	0.519(Cd) + 1.04	0.73	0.506(Cd) + 0.899	0.71	−2.27(Cd) + 94.6	0.61	−2.19(Cd) + 100	0.48
Chromium (Cr)	0.443(Cr) + 2.73	0.35	0.412(Cr) + 2.16	0.32	−2.34(Cr) + 89.8	0.43	−2.13(Cr) + 97.0	0.32
Copper (Cu)	0.0569(Cu) + 2.44	0.47	0.0551(Cu) + 1.83	0.50	−0.256(Cu) + 88.8	0.41	−0.283(Cu) + 98.6	0.48
Lead (Pb)	0.287(Pb) + 1.26	0.65	0.290(Pb) + 0.925	0.69	−1.30(Pb) + 94.2	0.57	−1.39(Pb) + 102	0.57
Zinc (Zn)	0.0133(Zn) + 0.409	0.62	0.0125(Zn) + 0.435	0.64	−0.064(Zn) + 99.5	0.63	−0.063(Zn) + 105	0.60

a All linear regressions are highly significant (*p* <0.01).

b Multiple linear regression analyses using all five metals as predictor variables revealed that Cd and Zn tissue concentrations cumulatively explained 87% (*r*^2^ = 0.87) of the variability in micronuclei frequency (‰MF) for *X. securis*, for both the 2004 [‰MF = 0.362(Cd) + 0.0706(Zn) – 0.497] and 2019 [‰MF = 0.342(Cd) + 0.00698(Zn) – 0.217] sampling events.

c Multiple linear regression analyses using all five metals as predictor variables revealed that (a) Cd, Cr and Zn tissue concentrations cumulatively explained 82% (*r*^2^ = 0.82) of the variability in percentage lysosomal membrane stability (%LMS) in *X. securis* for the 2004 sampling event [%LMS = −1.18(Cd) – 0.818(Cr) - 0.035(Zn) + 104] and (b) Cr, Pb and Zn tissue concentrations cumulatively explained 72% (*r*^2^ = 0.72) of the variability in %LMS in *X. securis* for the 2019 sampling event [%LMS = −1.04(Cr) – 0.765(Pb) – 0.026(Zn) + 108].

**Fig. 6:**
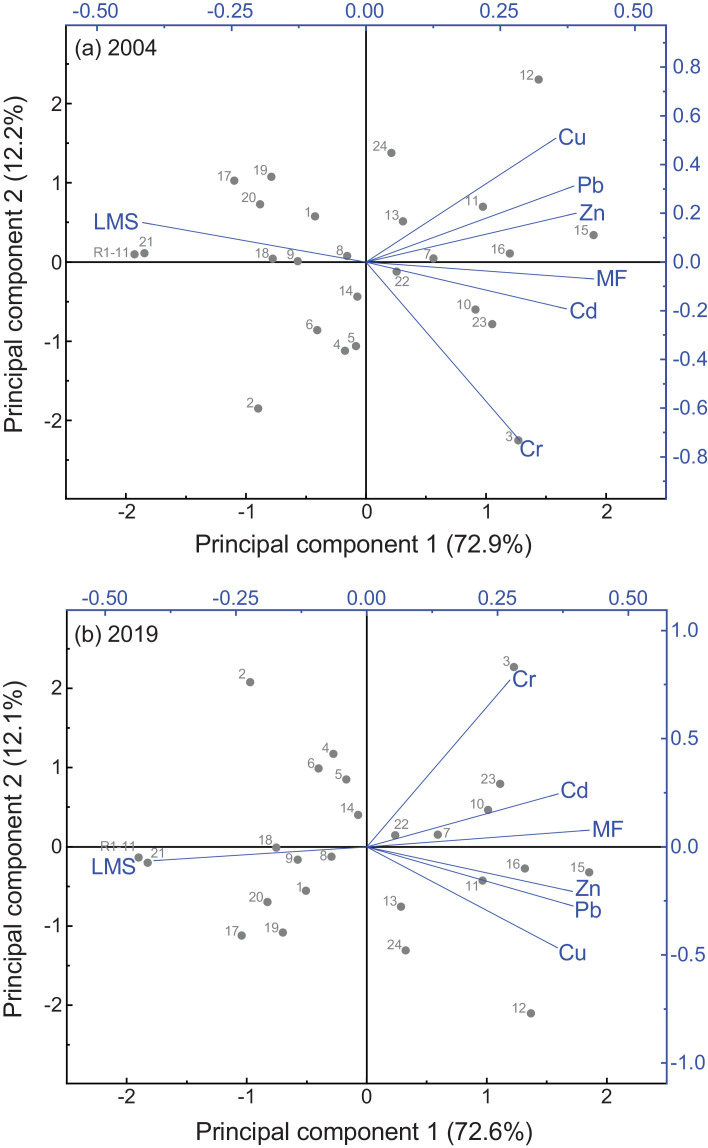
Two-dimensional principal components biplots showing site scores (gray circles) and loadings (or correlations) of the environmental variables (blue vector lines) from variably-contaminated sites (1–24) in the Sydney Estuary, and near-pristine (reference) sites (mean of R1−11) in the Hawkesbury Estuary, for (a) 2004 and (b) 2019. The environmental variables include cadmium (Cd), chromium (Cr), copper (Cu), lead (Pb) and zinc (Zn) tissue concentrations and toxicity effects (micronuclei frequency (MF) and percentage lysosomal membrane stability (LMS) in *Xenostrobus securis*. The first two principal components explain 85% of the variability (with the first component explaining the majority (73%) of the variance), for both sampling events. Two components provided the optimal model fit based on scree plots and/or parallel analyses (data not shown). The smaller the angle between two (blue) vector lines, the higher the correlation (e.g. Cd and MF in both plots, which is consistent with the linear regression (*r*^2^) results provided in Table 4). Inverse correlations are evident between LMS and all other variables in both plots. Site 3 shows separation from other sites, with Cr being a strong driver, while Zn is a strong driver for the separation of Site 15 from other sites. See Table S7 for the (i) coefficients of the two principal components and (ii) the percentage variance that can be explained for each environmental variable.

**Fig. 7:**
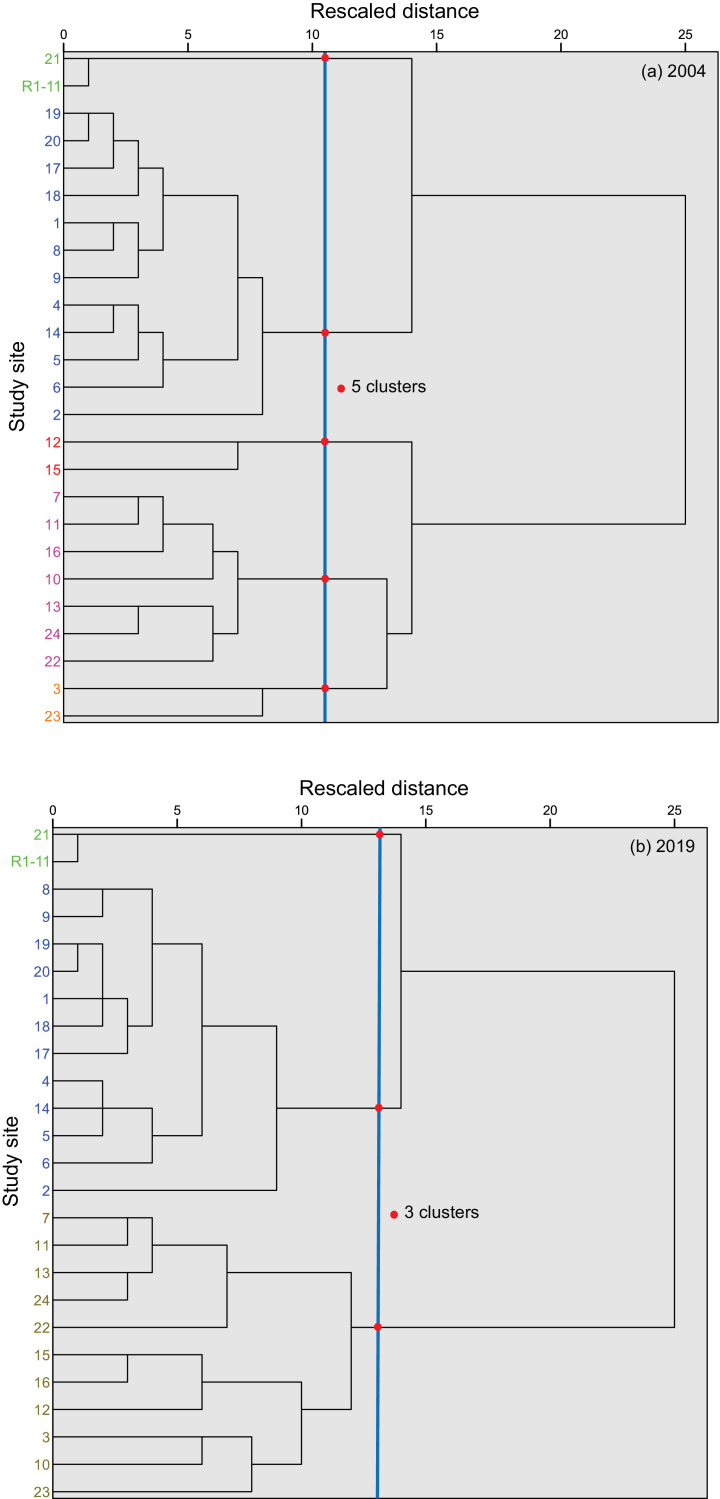
Dendrograms from agglomerative hierarchical cluster analysis, showing optimum clustering of sites (blue vertical line showing red dots at each key branch/cluster) similarly grouped according to cadmium, chromium, copper, lead and zinc tissue concentrations and toxicity effects (micronuclei frequency (‰) and percentage lysosomal membrane stability) in *Xenostrobus securis*, for (a) 2004 and (b) 2019. The optimum cluster number (and model validity/quality) was determined using the silhouette coefficient (SC) and normalized mutual information (NMI) - five clusters for 2004 with SC = 0.56 and NMI = 0.77, and three clusters for 2019 with SC = 0.59 and NMI = 0.81.

**Fig. 8:**
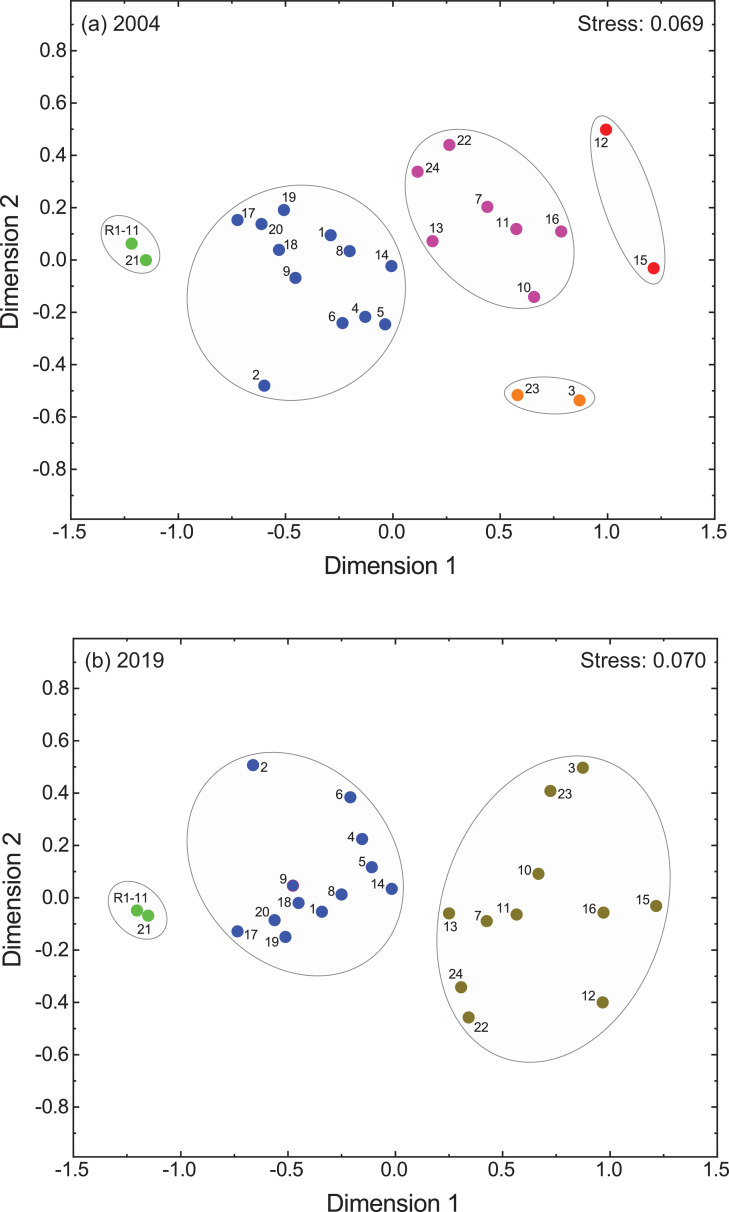
Two-dimensional non-metric multidimensional scaling ordination plots for the (a) 2004 and (b) 2019 sampling events. The smaller the distance between two objects (sites), the higher their similarity with respect to cadmium, chromium, copper, lead and zinc tissue concentrations and toxicity effects (micronuclei frequency (‰) and percentage lysosomal membrane stability) in *Xensotrobus securis*. Groups of similar sites, based on the results of cluster analysis (see Fig. 6) are shown in different colours (green, blue, pink orange or red for the 2004 sampling, and green, blue and brown for the 2019 sampling) and circled (in gray) on each ordination plot. The green sites (R1−11 and 21) represent the lowest metal bioaccumulation and toxicity, whereas the red/orange (2004) or brown (2019) sites (notably Sites 3, 12, 15 and 16) represent the highest metal bioaccumulation and toxicity. Note that sites within the green and blue clusters (or groupings) are consistent for both (2004 and 2019) sampling events, but sites within the pink, orange and red clusters for the 2004 sampling merge to form a new brown cluster in 2019 (indicating sites have become less dissimilar with respect to metal bioaccumulation and toxicity). The stress values of 0.069 (2004 sampling) and 0.070 (2019 sampling) indicate a good model fit (i.e. <0.15 for two dimensions, based on Dugard et al. [14], where the smaller the stress value, the better the model fit). Tuckers congruence coefficients (0.998 for both sampling events), or the square root of explained variance (*r*^2^), indicate that 99.5% of variance in the model is explained by the two dimensions (i.e. the variables can be represented in two dimensions with no doubt).

Two-dimensional principal component biplots, showing site scores (gray circles) and loadings (or correlations) of the environmental variables from variably-contaminated sites (1–24) in the Sydney Estuary and reference sites (R1−11) in the Hawkesbury Estuary, for the two sampling events (2004 and 2019), are presented in Fig. 6. The principal component coefficients and explained variance (%) for Cd, Cr, Cu, Pb and Zn tissue concentrations and the toxicity effects (‰MF and %LMS) in *X. securis*, are given in Appendix A (Table S7). Dendrograms from agglomerative hierarchical cluster analysis, showing sites similarly grouped according to Cd, Cr, Cu, Pb and Zn tissue concentrations and toxicity effects (‰MF or %LMS) in *X. securis*, for the two sampling events (2004 and 2019), are presented in Fig. 7. Two-dimensional non-metric multidimensional scaling ordination plots, for both sampling events, are displayed in Fig. 8.

## 2 Experimental design, materials, and methods

### 2.1 Study area – sampling sites, key contaminants and test species

The Sydney Estuary (Fig. 1), comprising the Parramatta and Lane Cove Rivers, and Middle and Sydney Harbours in south-eastern Australia, is a tide-dominated drowned river valley with a catchment area of 484 km^2^ (∼90% urbanized) and a length of 30 km. Tides are microtidal (mean and maximum tidal range is ∼1.0 m and 2.2 m, respectively) and mixed semi-diurnal. The surface waters are generally well mixed because of tidal turbulence and low freshwater inputs. Twenty-four sites (Fig. 1) were selected *a priori*, representing a wide range of chemical contamination, where wild populations of the suspension feeding pygmy mussel, *Xenostrobus securis* (Bivalvia: Mytilidae) (Lamarck 1819), resided in surface sediments [1,2]. In addition, 11 near-pristine sites surrounded by national parks with minimal urban influences, were selected in the lower reaches of the adjacent Hawkesbury Estuary (Fig. 1), which shares the same geology as the Sydney Estuary. These sites were used as reference (or background) sites for direct comparison with those in the Sydney Estuary.

For over two centuries, much of the Sydney Estuary has been consistently exposed to chemical contaminants from urban and industrial sources. While much of the existing chemical contamination in the Sydney Estuary is from legacy industrial point sources, catchment run-off (via stormwater drains and sewer overflows) and submarine groundwater discharge, are current and ongoing sources of chemical contamination. Five metals – Cd, Cr, Cu, Pb and Zn – were identified as key chemical contaminants in the Sydney Estuary, based on mean enrichment in *X. securis* [1].

*X. securis*, a synonym of *Limnoperna securis* and *L. fortunei kikuchii*, is endemic to the intertidal zone of estuaries and coastal lagoons of southern Australia (range 22°10′−43°28′ S) and New Zealand, but has been introduced to southeast Asia (Japan, China, South Korea) and southern Europe (France, Italy, Slovenia, Spain) in the last three decades (and now considered a problematic invasive species).

### 2.2 Mussel sampling and preparation

Wild populations of *X. securis* were sampled at each study site (Fig. 1) in June 2004 and 2019 to ascertain any change (over 15 years) in metal contaminant status (using whole soft tissue) and mussel health (genotoxicity: assessed via micronuclei frequency (MF) (Section 2.3), and cytotoxicity: assessed via lysosomal membrane stability (LMS) (Section 2.4)). About 200 mussels, covering the largest available size range (9−42 mm shell length), were collected from intertidal surface sediments (0−20 cm vertically above the mean low water mark) at each site. The byssal threads of individuals were carefully cut in situ with fine scissors. Shell length (the greatest anterior-posterior distance) was measured to the nearest 0.05 mm with Vernier calipers (Mitotoyo). Mussels were transported to the laboratory in insulated containers (in air at ambient temperature, typically within 4 h of collection) and depurated (to void gut contents) for 36 h in reconstituted seawater ([3]; salinity 30 ± 1‰, pH 7.8 ± 0.1, 16 ± 1 °C, see Tables 1 and 2) under flow-through conditions (95% replacement every 12 h) with a 14 h dark/10 h light (30 µmol m^−2^/s) photoperiod.

Following depuration, 20 mussels, comprising the largest 15% of the population (i.e. 21−24 months old, corresponding to 36−42 mm shell length at reference sites) from each site, were assessed for (i) MF (Section 2.3), (ii) LMS (Section 2.4) and (iii) whole soft tissue concentrations of Cd, Cr, Cu, Pb and Zn (Section 2.5). All mussels were collected early in their reproductive cycle (i.e. non-spawning phase); microscopic examination (x100 magnification) of mantle smears revealed that gametogenesis had commenced, but ripe gametes were not visible. There was no evidence of disease from parasites or pathogens in the tissues of sampled mussels. There were no significant (*p* >0.05) differences in MF, or LMS, between individuals (*n* = 10 per site) acclimated in the laboratory (for 36 h) and collected from the field (based on Sites 2, 3, 6, 15, 22 and R5, that encompassed the full range of contaminant exposure).

The haemocytes of *X. securis* were used to evaluate the genotoxicity and cytotoxicity of chemical contaminants (primarily Cd, Cr, Cu, Pb and Zn) in the Sydney Estuary. Haemocytes, which form a large part of the haemolymph (or “blood”), are circulating single cells in an open vascular system that are exposed to contaminants that enter the body via food (digestive glands) and/or the water column (gills). They are involved in the circulation of oxygen and nutrients and play an important role in immune defense (e.g. phagocytosis) and the transport, accumulation (detoxification) and excretion of contaminants [4].

### 2.3 Micronuclei frequency (genotoxicity)

Micronuclei formation provides a cumulative (time-integrated) and irreversible measure of chromosomal DNA damage (and genetic instability) caused by chromosome breakage or mis-segregation during mitosis [5]. Although micronuclei formation occurs naturally at very low frequencies (∼1 in every 1000 cells on average; see Appendix A (Table S1)) in marine mussels from minimally-contaminated sites, enhanced contaminant (e.g. Cd, Cr, Cu, Pb and Zn) exposure is well known to increase micronuclei frequency in a dose-dependent manner (genotoxicity) over their lifetime. The MF test is one of the most simple and reliable measures of genotoxicity, and the only one currently used, or proposed, in international marine pollution monitoring programs [5].

For each mussel, 100 µL of haemolymph was extracted from the posterior adductor mussel sinus using a 1 mL sterile syringe (with a 22 gauge hypodermic needle), containing 100 µL of buffered saline (137 mM NaCl, 10 mM Na_2_HPO_4_, 2.7 mM KCl and 1.8 mM KH_2_PO_4_; pH 7.4), gently mixed in a siliconized Eppendorf tube (1.5 mL) and maintained at 4 °C. 80 µL of haemolymph suspension was gently spread on a glass microscope slide (pre-coated with 10% poly-l-lysine solution), placed in a dark humid chamber (17 °C) for 15 min for cells to adhere/settle, air-dried (room temperature, 20 °C) for 10 min, fixed with 100% methanol for 10 min and air-dried for a further 10 min. Haemocytes were then stained (5% (v/v) Giesma solution) for 10 min, rinsed twice in washing solution (96.9 mg/L KH_2_PO_4_ and 109 mg/L NaPO_4_·2H_2_O) for 5 min each time, mounted (Eukitt) and air-dried overnight. Slides were then coded and randomized.

For each mussel, duplicate slides of 1000 intact agranular haemocytes were scored blind for MF (expressed as ‰) using a light microscope (Olympus BX50) at x1000 magnification. Micronuclei were identified according to the following criteria: (i) spherical or ovoid-shaped extra nuclear, non-refractory, bodies in the cytoplasm, (ii) diameter 1/3‒1/16 of the main nucleus, (iii) color, texture, and optical features resembling the main nucleus, and (iv) separate from the main nucleus (absence of chromatin bridge). The mean ‰MF was calculated using 20 mussels per site.

Haemocyte viability was assessed using differential acridine orange/ethidium bromide (AO/EB) staining [6]. Briefly, 20 µL of haemolymph suspension was mixed with 2 µL AO/EB stain (2 µg/mL) and the viability of 100 cells (per slide) was evaluated using a fluorescence microscope (Olympus BX50) at x400 magnification (AO stains viable or live cells bright green and EB stains apoptotic/necrotic cells orange/red). Mean haemocyte viability was >95% for all reference sites (R1‒11; Fig. 1).

### 2.4 Lysosomal membrane stability (cytotoxicity)

The LMS of haemocytes is widely recognised as a good indicator of cellular integrity (and general organism stress/health) in marine mussels [7], and is currently used in international marine pollution monitoring programs [8]. The neutral red retention test (NRRT) provides a simple, rapid and sensitive measure of LMS, where the loss of red dye from the lysosome to the cytosol over time, in addition to lysosomal abnormalities (enlargement and rounding up of haemocytes), reflects membrane damage (and loss of lysosomal integrity). The NRRT used herein was adapted from Lowe et al. [9] and Marchi et al. [10].

For each mussel, 100 µL of haemolymph was extracted from the posterior adductor mussel sinus using a 1 mL sterile syringe (with a 22 gauge hypodermic needle), containing 100 µL of buffered saline (429 mM NaCl, 52 mM MgSO_4_, 9.8 mM KCl, 9.8 mM CaCl_2_ and 20 nM 4-(2-hydroxyethyl)−1-piperazine-ethanesulfonic acid (HEPES); pH 7.4; salinity 30‰), gently mixed in a siliconized Eppendorf tube (1.5 mL) and maintained at 4 °C for up 20 min. 40 µL of haemolymph suspension was gently spread on a glass microscope slide (pre-coated with 10% poly-l-lysine solution), incubated in a dark humid chamber (17 °C) for 15 min for cells to adhere/settle and stained with 40 µL of neutral red (NR) solution (0.2 mM; from a stock solution of 28.8 mg NR (Sigma) in 1 mL of dimethyl sulfoxide, 10 µL was added into 5 mL buffered saline) for 15 min. Slides were then coded and randomized.

For each mussel, duplicate slides of stained haemocytes were consecutively incubated for 15, 30, 60, 90 and 120 min and percentage lysosomal membrane stability (%LMS) was determined, using the scoring procedure developed by Martínez-Gómez et al. [7] (see Appendix A (Table S8) for examples of scoring and %LMS calculations), using a light microscope (Olympus BX50) at x400 magnification. For each of the above incubation times, haemocyte images were captured (randomly across ∼20 fields of view) using an integrated digital camera (Olympus DP50) and scored blind (using ∼200 intact granular haemocytes per slide). Digital images acquired from 2004 were retrospectively scored. The mean %LMS was calculated using 20 mussels per site.

The toxicity (effect) endpoint used herein provides a quantitative measure (0–100%) of LMS, based on NR dye loss or lysosomal abnormalities [7] in ≥50% of cells. This approach improves upon the standard semi-quantitative (step) approach typically used in studies on marine mussels that use NRRT (corresponding to the last time (15, 30, 60, 90, 120, 150 or 180 min) recorded when there was no evidence of NR dye loss or lysosomal abnormalities in ≥50% of cells), by providing increased sensitivity and a wider response range.

### 2.5 Soft tissue metal concentrations

The whole soft tissue of each mussel (20 per site) was carefully dissected from the shell (after haemolymph extraction; see Sections 2.3 and 2.4), thoroughly rinsed with deionised water, blotted dry (wet weight) and oven-dried (40 °C) to a constant measured weight (dry weight, to the nearest 0.0001 g). Each sample was finely ground to a homogenous powder using a Teflon coated mortar and pestle and then solubilised in 65% nitric acid (7 mL) and 30% hydrogen peroxide (2 mL) using a microwave digestion system (Milestone ETHOS 1). The resulting clear digest solution was cooled, filtered (Whatman No. 542) and volume adjusted (25 mL) with deionised water prior to metal analysis. The concentrations of Cd, Cu, Pb and Zn in mussel digest solutions were measured using inductively coupled plasma mass spectrometry (Agilent 4500 or 7900). Gallium, indium and rhenium were employed as internal standards to correct for any non-spectral interferences. The concentration of Cr in mussel digest solutions was measured using inductively coupled plasma atomic emission spectrometry (Varian Vista AX or Agilent 700).

All reagents used were analytical grade, except for ultrapure nitric acid (Normaton). All solutions were prepared with deionised water (Milli-Q, 18 MΩ/cm). Procedural blanks were employed throughout mussel digestions and analyses to evaluate contamination. All analyses were corrected for blanks. A standard reference material (SRM; Community Bureau of Reference mussel tissue 278R) and sample duplicates were used to evaluate analytical accuracy and precision, respectively. The mean measured concentrations of Cd, Cr, Cu, Pb and Zn in the SRM were within their certified ranges. For duplicate samples and SRMs, the percentage coefficient of variation was typically 5 − 10%.

### 2.6 Surface water physico-chemistry

Surface water (∼50 cm depth) at each site was measured in situ for salinity (‰), pH, temperature (°C), turbidity (NTU) and dissolved oxygen (% saturation), twice in June 2004 and 2019 during dry conditions, using a multiparameter water quality meter (YSI 6000UPG or Horiba U52). The pH was measured with a glass combined electrode calibrated using a tris/tris-HCl buffer (on a total pH scale) according to Del Valls and Dickson [11]. All other electrodes/probes were calibrated according to the manufacturer's instructions using appropriate standard solutions. Chlorophyll *a*, a proxy for pelagic (micro-) phytoplankton abundance (and an estimate of food levels for *X. securis*), in surface waters at each site was sampled and measured according to Markich and Jeffree [2].

### 2.7 Data analyses

Spatial (between sites) and temporal (between years) differences in (i) ‰MF, (ii) %LMS or (iii) whole soft tissue metal (Cd, Cr, Cu, Pb or Zn) concentrations were tested using factorial (two-way) analysis of variance. Where significant differences were detected, mean values were graphically compared, whereby non-overlapping 84% confidence intervals were deemed significantly (*p* ≤ 0.05) different [12]. Linear regression analyses were used to investigate relationships between whole soft tissue metal concentrations and (i) ‰MF or (ii) %LMS. The assumptions of analysis of variance and linear regression were tested, and model adequacy was confirmed in all cases using either raw or transformed (log_10_) data. Significance was tested at the *p* = 0.05 level.

Multivariate statistical techniques were used to simultaneous examine several (dependent and independent) variables to reduce dimensionality, recognize patterns and group similar data. Principal components analysis (PCA), based on singular value decomposition of the correlation matrix, was used to compare similarities among sites based on metal (Cd, Cr, Cu, Pb or Zn) tissue concentrations and toxicity effects (‰MF and %LMS) in *X. securis*, to maximize (and explain) variance. The number of principal components was determined using a scree plot and/or parallel analysis (using the 95th percentile rule). Non-metric multidimensional scaling (nMDS), based on squared Euclidean distance on standardized (−1 to 1) data with 1000 random starts as the initial configuration (employing the PROXSCAL algorithm [13] with no ties), was used to ordinate sites according to metal tissue concentrations and toxicity effects in *X. securis*, as an alternative to PCA. Goodness of fit was assessed using a Shepard diagram, stress (stress-I) and/or Tuckers congruence coefficient. Hierarchical cluster analysis, based on Euclidean distance and unweighted average-linkage agglomeration on standardized (−1 to 1) data, was subsequently used to group similar sites on the nMDS ordination plots. Optimum cluster validity/quality was assessed using a combination of cluster indices (cophenetic correlation, silhouette coefficient and normalized mutual information) and visual dendrogram inspection.

An enrichment factor (EF) approach was also used to normalize and quantify the level of metal contamination, whereby the mean concentration of Cd, Cr, Cu, Pb or Zn in the whole soft tissue in *X. securis* from a given site in the Sydney Estuary, was divided by its mean “background” concentration, pooled from mussels at 11 near-pristine sites in the adjacent Hawkesbury Estuary.

This dataset was designed to minimize, or account for, the effects of key environmental (salinity, pH, temperature, turbidity, dissolved oxygen concentration and chlorophyll *a* concentration in site surface waters, shoreline height for mussel collection, and photoperiod) and biological (mussel age and reproductive status) variables, and hence, maximize the capacity to discern the potential effects (geno- and cyto-toxicity and accumulation) of (key metal) contaminants on *X. securis*.

## Appendix Supplementary materials

Image, application 1
