# Supplementary material for: Dataset of genotoxic and cytotoxic effects on the pygmy mussel, Xenostrobus securis, from the highly urbanised Sydney Estuary, Australia: Relationships with metal bioaccumulation
Source: Data Brief. 2020 Mar 20;30:105460. doi: 10.1016/j.dib.2020.105460 (PMC7152702; doi:10.1016/j.dib.2020.105460)
Supplement: Supplementary file 1 [file mmc1.docx]

Dataset of genotoxic and cytotoxic effects on the pygmy mussel, *Xenostrobus securis*, from the highly urbanised Sydney Estuary, Australia: Relationships with metal bioaccumulation

Scott J. Markich ^a, b^

*^a^ Aquatic Solutions International, “Point Break”, North Narrabeen Beach, NSW 2101, Australia*

*^b^ Department of Environmental Sciences, Macquarie University, North Ryde, NSW 2109, Australia*

# Appendix A. Supplementary data

**Table S1**

Micronuclei frequency (‰) and percentage lysosomal membrane stability in haemocytes of *Xenostrobus securis* from near-pristine (reference) sites in the Hawkesbury Estuary, for two sampling events (2004 and 2019).

| Site^a^ | Micronuclei frequency (‰) | |  | Lysosomal membrane stability (%) | |
| --- | --- | --- | --- | --- | --- |
|  | 2004 | 2019 |  | 2004 | 2019 |
| R1 | 0.84 ± 0.078^b^ | 1.2 ± 0.084 |  | 98.6 ± 0.82 | 99.3 ± 0.80 |
| R2 | 0.46 ± 0.072 | 0.62 ± 0.072 |  | 97.7 ± 0.89 | 97.8 ± 0.87 |
| R3 | 0.65 ± 0.074 | 0.49 ± 0.076 |  | 99.5 ± 0.78 | 96.9 ± 0.93 |
| R4 | 0.62 ± 0.081 | 0.78 ± 0.074 |  | 98.6 ± 0.79 | 98.8 ± 0.84 |
| R5 | 0.35 ± 0.062 | 0.45 ± 0.070 |  | 98.1 ± 0.82 | 99.3 ± 0.81 |
| R6 | 0.94 ± 0.081 | 0.72 ± 0.076 |  | 97.4 ± 0.85 | 98.6 ± 0.85 |
| R7 | 1.1 ± 0.080 | 0.95 ± 0.079 |  | 99.8 ± 0.88 | 97.3 ± 0.89 |
| R8 | 0.55 ± 0.071 | 0.70 ± 0.076 |  | 99.5 ± 0.80 | 97.2 ± 0.88 |
| R9 | 0.72 ± 0.075 | 0.65 ± 0.075 |  | 98.4 ± 0.78 | 98.9 ± 0.84 |
| R10 | 0.77 ± 0.081 | 0.83 ± 0.077 |  | 97.6 ± 0.81 | 98.5 ± 0.83 |
| R11 | 0.39 ± 0.065 | 0.41 ± 0.069 |  | 98.2 ± 0.83 | 97.9 ± 0.88 |
| **Mean**^c^ | **0.63 ± 0.10** | **0.67 ± 0.10** |  | **98.5 ± 0.89** | **98.2 ± 0.93** |

^a^ See Fig. 1 for location map.

^b^ Mean ± 84% confidence limit (n=20).

^c^ There were no significant (*p* ≤0.05) differences in the pooled mean values for micronuclei frequency (‰) or percentage lysosomal membrane stability between 2004 and 2019.

**Table S2**

Micronuclei frequency (‰) and percentage lysosomal membrane stability in haemocytes of *Xenostrobus securis* from variably contaminated sites in the Sydney Estuary, for two sampling events (2004 and 2019).

| Site^a^ | Micronuclei frequency (‰) | |  | Lysosomal membrane stability (%) | |
| --- | --- | --- | --- | --- | --- |
|  | 2004 | 2019 |  | 2004 | 2019 |
| 1 | 3.63 ± 0.26^b^ | 2.85 ± 0.21 |  | 78.2 ± 1.89 | 93.8 ± 1.25 |
| 2 | 2.82 ± 0.21 | 2.26 ± 0.16 |  | 85.1 ± 1.61 | 97.9 ± 0.84 |
| 3^c^ | 10.9 ± 0.83 | 7.32 ± 0.54 |  | 56.8 ± 2.25 | 73.8 ± 1.91 |
| 4 | 3.65 ± 0.27 | 2.91 ± 0.22 |  | 71.6 ± 1.99 | 88.1 ± 1.47 |
| 5 | 6.01 ± 0.42 | 4.48 ± 0.32 |  | 72.6 ± 1.96 | 89.5 ± 1.48 |
| 6 | 4.89 ± 0.36 | 3.70 ± 0.27 |  | 81.6 ± 1.78 | 95.1 ± 1.22 |
| 7 | 7.57 ± 0.55 | 5.37 ± 0.42 |  | 79.1 ± 1.90 | 88.0 ± 1.51 |
| 8 | 5.19 ± 0.37 | 3.74 ± 0.28 |  | 71.4 ± 1.95 | 88.5 ± 1.46 |
| 9 | 3.21 ± 0.24 | 2.51 ± 0.17 |  | 79.8 ± 1.82 | 91.3 ± 1.32 |
| 10 | 9.16 ± 0.68 | 6.50 ± 0.52 |  | 61.2 ± 2.16 | 74.4 ± 1.92 |
| 11^c^ | 8.81 ± 0.67 | 6.08 ± 0.44 |  | 70.0 ± 2.01 | 83.6 ± 1.68 |
| 12^c^ | 8.32 ± 0.60 | 5.80 ± 0.44 |  | 57.6 ± 2.23 | 75.3 ± 1.86 |
| 13 | 7.05 ± 0.52 | 5.22 ± 0.38 |  | 71.5 ± 2.01 | 90.3 ± 1.45 |
| 14 | 4.62 ± 0.33 | 3.64 ± 0.27 |  | 68.8 ± 2.00 | 85.6 ± 1.58 |
| 15^c^ | 10.1 ± 0.76 | 6.84 ± 0.50 |  | 49.2 ± 2.32 | 66.1 ± 2.02 |
| 16^c^ | 9.68 ± 0.71 | 6.87 ± 0.52 |  | 53.2 ± 2.25 | 68.3 ± 1.95 |
| 17 | 2.10 ± 0.19 | 1.70 ± 0.15 |  | 92.5 ± 1.20 | 95.5 ± 1.08 |
| 18 | 3.89 ± 0.30 | 3.11 ± 0.26 |  | 85.9 ± 1.56 | 95.6 ± 1.12 |
| 19 | 4.13 ± 0.31 | 3.28 ± 0.28 |  | 89.5 ± 1.46 | 93.6 ± 1.22 |
| 20 | 3.21 ± 0.25 | 2.56 ± 0.20 |  | 90.6 ± 1.43 | 95.6 ± 1.15 |
| 21 | 0.76 ± 0.11 | 0.80 ± 0.11 |  | 97.4 ± 0.91 | 98.9 ± 0.82 |
| 22 | 6.05 ± 0.44 | 4.50 ± 0.34 |  | 65.3 ± 2.07 | 80.2 ± 1.84 |
| 23^c^ | 6.78 ± 0.52 | 4.92 ± 0.38 |  | 61.5 ± 2.10 | 76.2 ± 1.88 |
| 24 | 5.96 ± 0.44 | 4.53 ± 0.34 |  | 80.8 ± 1.86 | 91.6 ± 1.29 |

^a^ See Fig. 1 for location map.

^b^ Mean ± 84% confidence limit (n=20).

^c^ There was a 30% decrease (*p* ≤0.05) in micronuclei frequency (‰), and a 16% increase in percentage lysosomal membrane stability, between 2004 and 2019, for the six most contaminated sites (based on combined metal enrichment factors in *X.* *securis*; see Fig. 5 and Table S6).

**Table S3**

Micronuclei frequency (‰) in haemocytes of wild mussels (Mytilidae) from minimally-contaminated (reference) sites (salinity >25‰)^a^.

| Species | Location  (Hemisphere)^b^ | Micronuclei  frequency (‰)^c^ | Reference |
| --- | --- | --- | --- |
| *Mytilus edulis* | Denmark (N) | 1.0 | [1] |
|  | United Kingdom (N) | 1.1 | [2] |
|  | United Kingdom (N) | 2.6 | [3] |
|  | Norway (N) | 1.8 | [4] |
| *Mytilus galloprovincialis* | Italy (N) | 1.4 | [5] |
|  | Greece (N) | 1.0 | [6] |
|  | Italy (N) | 0.80 | [7] |
|  | Italy (N) | 2.0 | [8] |
|  | Italy (N) | 1.2 | [9] |
|  | Croatia (N) | 1.5 | [10] |
|  | Portugal (N) | 0.63 | [11] |
|  | United Kingdom (N) | 1.7 | [12] |
|  | Italy (N) | 0.50 | [13] |
|  | Algeria (N) | 3.2 | [14] |
|  | Turkey (N) | 1.1 | [15] |
|  | Turkey (N) | 1.1 | [16] |
|  | Italy (N) | 0.36 | [17] |
| *Perna perna* | Brazil (S) | 0.80 | [18] |
| *Perna viridis* | China (N) | 0.32 | [19] |
|  | Singapore (N) | 1.4 | [20] |
| *Perumytilus purpuratus* | Chile (S) | 1.8 | [21] |
| *Xenostrobus securis* | Australia (S) | 0.65^d^ | This study |
| **Mean (range)** |  | **1.1 (0.32−3.2)** |  |

^a^ Excludes farmed mussels.

^b^ N = Northern hemisphere (86% of studies), S = Southern hemisphere (14% of studies).

^c^ Mean values.

^d^ See Table S1 for raw data. The mean micronuclei frequency (‰) for haemocytes of *X. securis* from the Hawkesbury Estuary occurs within the range of other mytilid bivalves from minimally-contaminated sites worldwide.

**Table S4**

Summary table for results of factorial (two-way) analysis of variance, with study site (1−24 and R1−11) and sampling time (2004 or 2019) as independent variables and micronuclei frequency (‰) in the haemocytes of *Xenostrobus securis* as the dependent variable^a^.

|  | DF^b^ | Sum of Squares | Mean Square | *F* value | *p* value |
| --- | --- | --- | --- | --- | --- |
| Study site | 24 | 268.9 | 11.21 | 22.07 | <0.0001^c^ |
| Sampling time | 1 | 27.32 | 27.32 | 53.81 | <0.0001^d^ |
| Model | 25 | 296.2 | 11.85 | 23.34 | <0.0001 |
| Error | 24 | 12.18 | 0.5077 |  |  |
| Corrected total | 49 | 308.4 |  |  |  |

^a^ The interaction between study site and sampling time was not significant (*p* =0.084).

^b^ DF, degrees of freedom.

^c^ The population means are significantly (*p* ≤0.05) different. Fig. 2 shows where significant differences occur among study sites (i.e. non-overlapping confidence intervals) for a given sampling time.

^d^ The population means are significantly (*p* ≤0.05) different. Fig. 2 shows where significant differences occur between sampling times (i.e. non-overlapping confidence intervals) for a given study site.

**Table S5**

Summary table for results of factorial (two-way) analysis of variance, with study site (1−24 and R1−11) and sampling time (2004 or 2019) as independent variables and percentage lysosomal membrane stability in the haemocytes of *Xenostrobus securis* as the dependent variable^a^.

|  | DF^b^ | Sum of Squares | Mean Square | *F* value | *p* value |
| --- | --- | --- | --- | --- | --- |
| Study site | 24 | 6417 | 267.4 | 17.80 | <0.0001^c^ |
| Sampling time | 1 | 1871 | 1871 | 124.6 | <0.0001^d^ |
| Model | 25 | 8289 | 331.6 | 22.07 | <0.0001 |
| Error | 24 | 360.5 | 15.02 |  |  |
| Corrected total | 49 | 8649 |  |  |  |

^a^ The interaction between study site and sampling time was not significant (*p* =0.13).

^b^ DF, degrees of freedom.

^c^ The population means are significantly (*p* ≤0.05) different. Fig. 3 shows where significant differences occur among study sites (i.e. non-overlapping confidence intervals) for a given sampling time.

^d^ The population means are significantly (*p* ≤0.05) different. Fig. 3 shows where significant differences occur between sampling times (i.e. non-overlapping confidence intervals) for a given study site.

**Table S6**

Mean enrichment factors of cadmium, chromium, copper, lead and zinc in the whole soft tissue of *Xenostrobus securis* from variably contaminated sites in the Sydney Estuary, for two sampling events (2004 and 2019)^a^.

| Site^b^ |  | Cadmium | |  | Chromium | |  | Copper | |  | Lead | |  | Zinc | |
| --- | --- | --- | --- | --- | --- | --- | --- | --- | --- | --- | --- | --- | --- | --- | --- |
|  |  | 2004 | 2019 |  | 2004 | 2019 |  | 2004 | 2019 |  | 2004 | 2019 |  | 2004 | 2019 |
| 1 |  | 10.8 | 8.04 |  | 2.89 | 2.39 |  | 8.14 | 5.98 |  | 6.99 | 5.10 |  | 7.63 | 5.92 |
| 2 |  | 6.88 | 5.36 |  | 9.65 | 7.75 |  | 2.47 | 2.01 |  | 2.73 | 2.10 |  | 4.04 | 3.34 |
| 3 |  | 21.7 | 14.0 |  | 13.7 | 10.1 |  | 8.16 | 6.36 |  | 11.1 | 8.29 |  | 10.7 | 7.95 |
| 4 |  | 14.8 | 9.97 |  | 7.50 | 5.79 |  | 3.46 | 2.71 |  | 8.21 | 5.91 |  | 7.61 | 5.59 |
| 5 |  | 10.6 | 7.93 |  | 8.49 | 5.83 |  | 6.56 | 5.49 |  | 7.30 | 5.24 |  | 6.53 | 5.00 |
| 6 |  | 18.0 | 13.6 |  | 5.22 | 4.14 |  | 3.69 | 3.08 |  | 5.28 | 4.17 |  | 5.88 | 4.74 |
| 7 |  | 19.0 | 12.7 |  | 6.94 | 5.28 |  | 8.00 | 6.37 |  | 18.3 | 12.1 |  | 8.39 | 6.72 |
| 8 |  | 8.62 | 6.36 |  | 4.36 | 3.35 |  | 5.11 | 3.70 |  | 7.85 | 5.50 |  | 10.5 | 7.69 |
| 9 |  | 3.30 | 2.57 |  | 5.62 | 4.33 |  | 5.65 | 4.80 |  | 7.05 | 5.29 |  | 8.79 | 6.91 |
| 10 |  | 25.2 | 16.0 |  | 6.83 | 5.12 |  | 10.1 | 8.11 |  | 10.5 | 8.10 |  | 9.37 | 7.69 |
| 11 |  | 18.7 | 12.3 |  | 5.87 | 4.95 |  | 12.2 | 9.07 |  | 15.5 | 10.7 |  | 12.9 | 9.75 |
| 12 |  | 15.9 | 10.3 |  | 4.49 | 3.60 |  | 22.4 | 15.9 |  | 19.7 | 12.8 |  | 13.0 | 10.1 |
| 13 |  | 13.1 | 8.73 |  | 4.51 | 3.43 |  | 8.96 | 7.60 |  | 12.3 | 8.81 |  | 9.94 | 8.22 |
| 14 |  | 13.1 | 9.53 |  | 5.86 | 4.58 |  | 5.21 | 4.36 |  | 11.2 | 7.82 |  | 6.69 | 5.53 |
| 15 |  | 20.1 | 13.5 |  | 9.70 | 6.86 |  | 17.4 | 12.9 |  | 16.8 | 11.6 |  | 16.7 | 11.8 |
| 16 |  | 18.0 | 11.8 |  | 7.01 | 5.17 |  | 11.4 | 9.30 |  | 17.5 | 11.9 |  | 10.4 | 8.67 |
| 17 |  | 2.10 | 1.65 |  | 1.51 | 1.31 |  | 5.65 | 4.54 |  | 5.97 | 4.83 |  | 7.33 | 5.61 |
| 18 |  | 7.81 | 6.00 |  | 3.76 | 3.13 |  | 4.43 | 3.71 |  | 8.12 | 6.41 |  | 4.54 | 3.43 |
| 19 |  | 6.42 | 4.79 |  | 1.51 | 1.34 |  | 8.97 | 7.13 |  | 5.97 | 4.59 |  | 5.94 | 4.55 |
| 20 |  | 8.06 | 6.14 |  | 1.86 | 1.58 |  | 6.87 | 5.32 |  | 4.98 | 3.58 |  | 6.46 | 5.28 |
| 21 |  | 1.14 | 1.06 |  | 1.22 | 1.06 |  | 1.27 | 1.19 |  | 1.21 | 1.14 |  | 1.69 | 1.64 |
| 22 |  | 21.6 | 13.7 |  | 3.68 | 3.10 |  | 4.58 | 3.93 |  | 16.2 | 11.0 |  | 5.08 | 4.04 |
| 23 |  | 13.8 | 9.90 |  | 13.1 | 9.57 |  | 15.6 | 12.3 |  | 12.5 | 8.95 |  | 11.7 | 8.81 |
| 24 |  | 15.8 | 11.8 |  | 2.97 | 2.39 |  | 13.2 | 10.2 |  | 14.2 | 10.9 |  | 7.89 | 6.04 |

^a^ To calculate the enrichment factor (EF), the mean concentration of cadmium, chromium, copper, lead or zinc in the whole soft tissue in *X. securis* from a given study site in the Sydney Estuary, was divided by its mean “background” concentration (pooled from mussels at 11 near-pristine sites) in the adjacent Hawkesbury Estuary (e.g. the EF for zinc at site 1 (2019) is 277/46.8 = 5.92; see Table 3).

^b^ See Fig. 1 for location map.

**Table S7**

Principal component coefficients and explained variance (%) for metal tissue concentrations in *Xenostrobus securis* and two toxicity endpoints (micronuclei frequency (MF) and percentage lysosomal membrane stability (LMS).

|  | Principal component 1 | Principal component 2 | Variance explained (%)^a^ |
| --- | --- | --- | --- |
| **2004 sampling** |  |  |  |
| Cadmium | 0.373 | -0.192 | 75.0 |
| Chromium | 0.286 | -0.732 | 86.8 |
| Copper | 0.353 | 0.508 | 85.3 |
| Lead | 0.387 | 0.312 | 84.4 |
| Zinc | 0.391 | 0.200 | 81.0 |
| MF^a^ | 0.424 | -0.070 | 92.5 |
| LMS^b^ | -0.415 | 0.162 | 88.3 |
| **2019 sampling** |  |  |  |
| Cadmium | 0.367 | 0.244 | 73.3 |
| Chromium | 0.274 | 0.771 | 88.1 |
| Copper | 0.365 | -0.467 | 86.0 |
| Lead | 0.394 | -0.274 | 85.4 |
| Zinc | 0.394 | -0.206 | 82.4 |
| MF^a^ | 0.426 | 0.077 | 92.7 |
| LMS^b^ | -0.407 | -0.064 | 84.7 |

^a^ Variance (%) explained by two principal components (dimensions).

**Table S8**

Example calculations of percentage lysosomal membrane stability (%LMS) in *Xenostrobus securis* at two variably contaminated sites (Sites 16 and 24) in the Sydney Estuary, for two sampling events (2004 and 2019).

| Site/year | Time  (mins)^a^ | 15 |  |  | 30 |  |  | 60 |  |  | 90 |  |  | 120 |  |  | Sum of  weighted  scores | %LMS^b^ |
| --- | --- | --- | --- | --- | --- | --- | --- | --- | --- | --- | --- | --- | --- | --- | --- | --- | --- | --- |
|  | Weighting factor | 1 |  |  | 2 |  |  | 3 |  |  | 4 |  |  | 5 |  |  |  |  |
|  | Mussel/slide | NRR^c^ | Score^d^ | Wtd  score^e^ | NRR | Score | Wtd  score | NRR | Score | Wtd  score | NRR | Score | Wtd  score | NRR | Score | Wtd  score |  |  |
| 2/2004 | 1/a | + | 0 | 0 | + | 0 | 0 | + | 0 | 0 | ̶ | 1 | 4 | ̶ | 1 | 5 | 9 | 88.0^f^ |
|  | 1/b | + | 0 | 0 | + | 0 | 0 | ̶ | 1 | 3 | ̶ | 1 | 4 | ̶ | 1 | 5 | 12 | 84.0 |
|  | 2/a | + | 0 | 0 | + | 0 | 0 | + | 0 | 0 | ̶ | 0 | 0 | ̶ | 2 | 5 | 10 | 86.7 |
|  | 2/b | + | 0 | 0 | + | 0 | 0 | + | 0 | 0 | ̶ | 0 | 0 | ̶ | 2 | 5 | 10 | 86.7 |
| 2/2019 | 1/a | + | 0 | 0 | + | 0 | 0 | + | 0 | 0 | ̶ | 0 | 0 | ̶ | 1 | 5 | 5 | 93.3 |
|  | 1/b | + | 0 | 0 | + | 0 | 0 | + | 0 | 0 | ̶ | 0 | 0 | ̶ | 0 | 0 | 0 | 100 |
|  | 2/a | + | 0 | 0 | + | 0 | 0 | + | 0 | 0 | ̶ | 0 | 0 | ̶ | 0 | 0 | 0 | 100 |
|  | 2/b | + | 0 | 0 | + | 0 | 0 | + | 0 | 0 | ̶ | 0 | 0 | ̶ | 1 | 5 | 5 | 93.3 |
| 16/2004 | 1/a | + | 0 | 0 | + | 0 | 0 | ̶ | 2 | 6 | ̶ | 2 | 8 | ̶ | 5 | 25 | 39 | 48.0 |
|  | 1/b | + | 0 | 0 | + | 0 | 0 | + | 0 | 0 | ̶ | 2 | 8 | ̶ | 5 | 25 | 33 | 56.0 |
|  | 2/a | + | 0 | 0 | + | 0 | 0 | + | 0 | 0 | ̶ | 4 | 16 | ̶ | 4 | 20 | 36 | 52.0 |
|  | 2/b | + | 0 | 0 | ̶ | 1 | 2 | ̶ | 1 | 3 | ̶ | 3 | 15 | ̶ | 4 | 20 | 37 | 50.7 |
| 16/2019 | 1/a | + | 0 | 0 | ̶ | 1 | 2 | ̶ | 1 | 3 | ̶ | 1 | 4 | ̶ | 3 | 15 | 24 | 68.0 |
|  | 1/b | + | 0 | 0 | + | 0 | 0 | ̶ | 1 | 3 | ̶ | 1 | 4 | ̶ | 3 | 15 | 22 | 70.7 |
|  | 2/a | + | 0 | 0 | + | 0 | 0 | ̶ | 2 | 6 | ̶ | 2 | 8 | ̶ | 2 | 10 | 24 | 68.0 |
|  | 2/b | + | 0 | 0 | + | 0 | 0 | ̶ | 2 | 6 | ̶ | 2 | 8 | ̶ | 2 | 10 | 24 | 68.0 |

^a^ Exposure (incubation) time of haemocytes to neutral red stain (dye) for consecutive scoring of pathologies (see (d)).

^b^ %LMS = (1 - (sum of weighted scores/75)) x 100. As an example, the calculation of %LMS for mussel 1 (slide a) at Site 1(2004 sampling) = (1 - (9/75)) x 100 = 88%.

^c^ NNR, neutral red (dye) retention. Designated as + (plus sign) for a clear cytosol and no evidence of lysosomal abnormalities in ≥50% of cells, or – (minus sign) for evidence of dye loss and/or lysosomal abnormalities in ≥50% of cells.

^d^ Lysosomal pathology score (increasing severity of effect) for ≥50% of cells: 0 = no effect, 1 = enlargement but no leakage, 2 = leakage but no enlargement, 3 = leakage and enlargement, 4 = leakage and enlargement but colorless lysosomes, 5 = rounded up fragmenting cells.

^e^ Weighted (Wtd) score was calculated by multiplying the score by the weighting factor at each time.

^f^ Mean value of two slides per mussel, with the final mean %LMS based on 20 mussels per site (e.g. Site 2 (2004), mussel 1: slide a (88%) + slide b (84%) = 86% (mean).

# References

[1] M.N. Wrisberg, C.M. Bilbo, H. Spliid, Induction of micronuclei in hemocytes of *Mytilus edulis* and statistical analysis, Ecotox. Environ. Safe. 23 (1992) 191–205.

[2] J.A. Hagger, M.H. Depledge, T.S. Galloway, Toxicity of tributyltin in the marine mollusc *Mytilus edulis*, Mar. Pollut. Bull. 51 (2005) 811–816.

[3] M.N. Canty, T.H. Hutchinson, R.J. Brown, M.B. Jones, A.N. Jha, Linking genotoxic responses with cytotoxic and behavioural or physiological consequences: Differential sensitivity of echinoderms (*Asterias rubens*) and marine molluscs (*Mytilis edulis*), Aquat. Toxicol. 94 (2009) 68–76.

[4] S.J. Brooks, C. Esudero-Oñate, T. Gomes, L. Ferrando-Climent, An integrative biological effects assessment of a mine discharge into a Norwegian fjord using field transplanted mussels, Sci. Total Environ. 644 (2018) 1056–1069.

[5] L. Docetti. P. Vernier, Susceptibility to genetic damage and cell types in Mediterranean mussels, Mar. Environ. Res. 54 (2002) 487–491.

[6] S. Dailianis, G.P. Domouhtsidou, E. Raftopoulou, M. Kaloyianni, V.K. Dimitriadis, Evaluation of neutral red retention assay, micronucleus test, acetylcholinesterase activity and a signal transduction molecule (cAMP) in tissues of *Mytilus galloprovincialis* (L.), in pollution monitoring, Mar. Environ. Res. 56 (2003) 443–470.

[7] D.M. Pampanin, I. Marangon, E. Volpato, G. Campesan, C. Nasci, Stress biomarkers and alkali-labile phosphate level in mussels (*Mytilus galloprovincialis*) collected in the urban area of Venice (Venice Lagoon, Italy), Environ. Pollut. 136 (2005) 103–107.

[8] P. Venier, C. Zampieron, Evidence of genetic damage in grass gobies and mussels from the Venice Lagoon, Environ. Int. 31(2005) 1053–1064.

[9] S. Gorbi, V.L. Virno, C. Notti, M. Benedetti, D. Fattorini, G. Moltedo, F. Regoli, An ecotoxicological protocol with caged mussels, *Mytilus galloprovincialis*, for monitoring the impact of an offshore platform in the Adriatic Sea, Mar. Environ. Res. 65 (2008) 34–49.

[10] A. Štambuk, M. Šrut, Z. Šatović, M. Tkalec, G. I. Klobuĉar, Gene flow v pollution pressure: Genetic diversity of *Mytilus galloprovincialis* in eastern Adriatic, Aquat. Toxicol. 136–137 (2013) 22–31.

[11] T.L. Rocha, T. Gomes, C. Cardosa, J. Letendre, J.P. Pinheiro, V.S. Sousa, M.R. Teixeira, Immunocytotoxicity, cytogenotoxicity and genotoxicity of cadmium-based quantum dots in the marine mussel *Mytilus galloprovincialis*, Mar. Environ. Res. 101 (2014) 29–37.

[12] E. Barón, A. Dissanayake, J. Vila, C. Crowther, J.W. Readman, A. Jha, E. Eljarrat, D. Barcelo, Evaluation of the genotoxic and physiological effects of decabromodiphenyl (BDE-209) and dechlorane plus (DP) flame retardants in marine mussels (*Mytilus galloprovincialis*), Environ. Sci. Technol. 50 (2016) 2700–2708.

[13] V. Matozzo, V. Bertin, M. Battistara, A. Guidolin, L. Masiero, I. Marisa, A. Orsetti, Does the antibiotic amoxicillin affect haemocyte parameters in non-target aquatic invertebrates? The clam *Ruditapes phillippinarum* and the mussel *Mytilus galloprovincialis* as model organisms, Mar. Environ. Res. 119 (2016) 51–58.

[14] H.G. Touahri, Z. Boutiba, W. Benguedda, S. Shaposhnikov, Active biomonitoring of mussels *Mytilus galloprovincialis* with integrated use of micronucleus assay and physiological indices to assess harbor pollution, Mar. Pollut. Bull. 110 (2016) 52–64.

[15] B. Nalbantlar, O.C. Arslan, Determination of the perflooroctane sulfonate-induced genotoxic response in *Mytilus galloprovincialis* using a micronucleus assay, Zool. Ecol. 27 (2017) 161–167.

[16] D. Ozkan, M. Dagdeviren, S. Katalay, A. Guner, Multi-biomarker responses after exposure to pollution in the Mediterranean mussels (*Mytilus galloprovincialis* L.) in the Aegean coast of Turkey, Bull. Environ. Contam. Toxicol. 98 (2017) 46–52.

[17] M.E. Giuliani, S. Accoroni, M. Mezzelani, F. Lugarini, S. Bacchiocchi, M. Siracusa, T. Tavoloni, A. Piersanti, C. Totti, F. Regoli, R. Rossi, A. Zingone, S. Gorbi, 2019. Biological effects of the azaspiracid-producing dinoflagellate *Azadinium dexteroporum* in *Mytilus galloprovincialis* from the Mediterranean Sea, Mar. Drugs 17, 595.

[18] J.M. Gutiérrez, M.B. da Conceição, M.M. Olisani, L.I. Weber, Genotoxicity biomonitoring along a coastal zone under influence of offshore petroleum exploration (southeastern Brazil), Bull. Environ. Contam. Toxicol. 100 (2018) 338–343.

[19] W.H. Siu, J. Cao, R.W. Jack, R.S. Wu, B.J. Richardson, L. Xu, P.K. Lam, Application of the comet and micronucleus assays to the detection of B[*a*]P genotoxicity in haemocytes of the green-lipped mussel (*Perna viridis*), Aquat. Toxicol. 66 (2004) 381–392.

[20] C. Liu, V.W. Chang, K.Y. Gin, V.T. Nguyen, Genotoxicity of perfluorinated chemicals (PFCs) to the green mussel (*Perna viridis*), Sci. Total. Environ. 487 (2014) 117–122.

[21] H. Gaete, R. Guerra, P. Espinoza, D. Fernández, Lysosomal membrane stability in haemocytes and micronuclei in gills of *Perumytilus purpuratus* Lamarck 1819 (Bivalvia: Mytilidae) exposed to copper, Bull. Environ. Contam. Toxicol. 103 (2019) 796–801.
